# Supplementary figures and images for: Overexpressed PLAU and its potential prognostic value in head and neck squamous cell carcinoma
Source: PeerJ. 2021 Jan 15;9:e10746. doi: 10.7717/peerj.10746 (PMC7812932; doi:10.7717/peerj.10746)

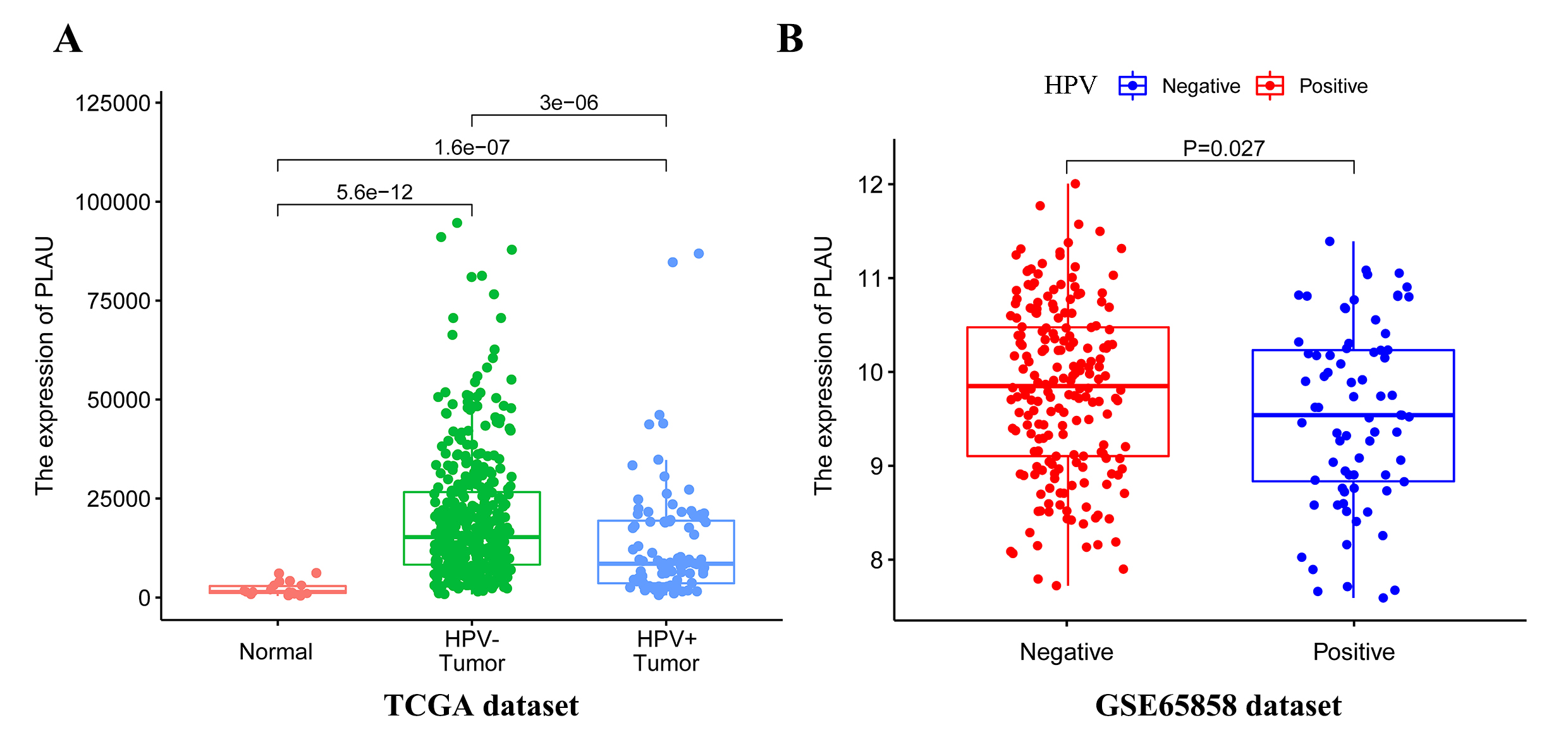

Supplement: Supplemental Information 17 — (A) Separating the groups by HPV status, the expression of PLAU mRNA compared with the normal samples were evaluated in HNSCC samples of TCGA databases. (B) The PLAU expression in HPV- and HPV+ of HNSCC patients. [file peerj-09-10746-s017.jpg]
